# Willingness to Share Research Data Is Related to the Strength of the Evidence and the Quality of Reporting of Statistical Results

**DOI:** 10.1371/journal.pone.0026828

**Published:** 2011-11-02

**Authors:** Jelte M. Wicherts, Marjan Bakker, Dylan Molenaar

**Affiliations:** Psychology Department, Faculty of Social and Behavioral Sciences, University of Amsterdam, Amsterdam, The Netherlands; Georgetown University Medical Center, United States of America

## Abstract

**Background:**

The widespread reluctance to share published research data is often hypothesized to be due to the authors' fear that reanalysis may expose errors in their work or may produce conclusions that contradict their own. However, these hypotheses have not previously been studied systematically.

**Methods and Findings:**

We related the reluctance to share research data for reanalysis to 1148 statistically significant results reported in 49 papers published in two major psychology journals. We found the reluctance to share data to be associated with weaker evidence (against the null hypothesis of no effect) and a higher prevalence of apparent errors in the reporting of statistical results. The unwillingness to share data was particularly clear when reporting errors had a bearing on statistical significance.

**Conclusions:**

Our findings on the basis of psychological papers suggest that statistical results are particularly hard to verify when reanalysis is more likely to lead to contrasting conclusions. This highlights the importance of establishing mandatory data archiving policies.

## Introduction

Statistical analyses of research data are quite error prone [1], [2], [3], accounts of statistical results may be inaccurate [4], and decisions that researchers make during the analytical phase of a study may lean towards the goal of achieving a preferred (significant) result [5], [6], [7], [8]. For these and other (ethical) reasons [9], many scientific journals like *PLoS ONE* [10] and professional organizations such as the *American Psychological Association* (APA) [11] have clear policies concerning the sharing of data after research results are published. For instance, upon acceptance for publication of a paper in one of the over 50 peer-reviewed journals published by the APA, authors sign a contract that they will make available data to peers who wish to reanalyze their data to verify the substantive claims put forth in the paper. Nonetheless, the replication of statistical analyses in published psychological research is hampered by psychologists' pervasive reluctance to share their raw data [1], [12]. In a large-scale study Wicherts et al. [12] found that 73% of psychologists publishing in four top APA journals defied APA guidelines by not sharing their data for reanalysis. The unwillingness to share data of published research has been documented in a number of fields [13], [14], [15], [16], [17], [18], [19], [20] and is often ascribed in part to the fear among authors that independent reanalysis will expose statistical or analytical errors in their work [21] and will produce conclusions that differ from theirs [22]. However, no published research to date has addressed whether this rather bleak scenario has a bearing on reality.

Here we study whether researchers' willingness to share data for reanalysis is associated with the strength of the evidence (defined as the statistical evidence against the null hypothesis of no effect) and the quality of the reporting of statistical results (defined in terms of the prevalence of inconsistencies in reported statistical results). To this end, we followed-up on Wicherts et al.'s requests for data [12] by comparing statistical results in papers from which data were either shared or not, and to check for errors in the reporting of p-values in both types of papers.

## Methods

In the summer of 2005, Wicherts and colleagues [12] contacted the corresponding authors of 141 papers that were published in the second half of 2004 in one of four high-ranked journals published by the APA: *Journal of Personality and Social Psychology (JPSP)*, *Developmental Psychology (DP)*, *Journal of Consulting and Clinical Psychology (JCCP)*, and *Journal of Experimental Psychology: Learning, Memory, and Cognition (JEP∶LMC)*. The data were requested to determine the effects of outliers on statistical outcomes (see Text S1 for details). Although all corresponding authors had signed a statement that they would share their data for such verification purposes [11], most authors failed to do so. In the current study, we related the willingness to share data from 49 papers published in *JPSP* or *JEP∶LMC* to two relevant characteristics of the statistical outcomes reported in the papers, namely the internal consistency of the statistical results and the distribution of significantly reported (*p*<.05) p-values. We restricted the attention to *JPSP* and *JEP∶LMC*, because (1) authors in these journals were more willing to share data than authors in the other journals from which Wicherts et al. requested data, (2) no corresponding authors in these two journals declined to share data, because they were part of an ongoing project or because of propriety rights or ethical considerations, and (3) studies in these two journals were fairly homogeneous in terms of analysis and design (mostly lab experiments). We also restricted our attention to results from null-hypothesis significance testing (NHST) [23]. This procedure is not without its critics [24], [25], but remains to be used extensively in psychology [26] and related fields. NHST provides p-values that, if smaller than alpha = .05, are considered by many researchers [27], [28] and reviewers [29] to lend support to the hypothesized effects. Psychological research data are often amenable to alternative methods of analysis [6], [22], [30] that may affect what can be concluded from them (at least within the rules of NHST). The specifics of the analysis will typically have more relevance when statistical results are nearly significant at the alpha = .05 level. Put differently, smaller p-values provide stronger evidence against the null hypothesis of no effect [31]. The strength of the evidence based on Bayes factors from Bayesian t-tests has been found to be strongly inversely related to the p-values of traditional t-tests [32]. If the strength of the evidence so defined plays part in the willingness to share data, then it is to be expected that p-values in papers from which data were not shared lie closer to .05. Because reported p-values are often inconsistent with the given test statistics and degrees of freedom [33], we also check for errors in reporting of statistical results.

### Data Retrieval

We extracted from the papers all the *t*, *F*, and *χ^2^* test statistics associated with NHST, the given degrees of freedom (e.g., *F*(2,24) = 3.41), the sidedness of tests (1- or 2-tailed), and the reported exact p-value (e.g., *p* = .03) or the reported level of significance (e.g., *p*<.05). We considered these tests because these are the most common test statistics of NHST in psychology. Although it was infeasible to determine for each test whether it was in line with the researchers' predictions, NHST is typically used for the purpose of rejecting the null hypothesis. We did not consider test statistics that were not associated with NHST (e.g., model fitting or Bayesian analyses). We only included test results that were uniquely reported, complete (i.e., test statistic, degrees of freedom, and p-value were reported), and that were reported as being significant (i.e., *p*<.05) in the main text or in tables in the results sections. T-tests were considered 2-tailed, unless stated otherwise. The exact p-values were computed on the basis of the given test statistic and DF(s) in Microsoft Excel 2008 for Mac, version 12.1.0. A further four papers published in the two journals from which data were requested in 2005 were not included in the follow-up because they did not involve NHST or did not contain significant results on the basis of *t*, *F*, of *χ^2^* tests.

Five undergraduates, who were unaware from which papers data were shared also independently retrieved a total of 495 statistics and DFs. We compared these 495 statistics to ours and determined that the accuracy rate in our own data was 99.4%. The three minor errors in our data retrieval were corrected but proved trivial.

### Detecting Reporting Errors

Inconsistencies between reported p-values (or ranges) and p-values recalculated from the retrieved statistics were detected automatically in Excel as follows. The recomputed p-value was first rounded to the same number of digits as was used in the reported p-value (range). Subsequently, an IF-statement automatically checked for consistency. Next, we determined by hand whether reporting errors were not due to possible errors in extraction (none were found) or to rounding. For example, a test result such as “t(15) = 2.3; p = 0.034” could have arisen from test statistic that could possibly range from 2.25 to 2.35. Consequently, the correct p-value could range from .033 to .040 and so the reported value was not seen as inconsistent, although the recomputed p-value is .0362. In the analyses of the p-value distributions, we used the nearest next decimal that attained consistency for these correctly rounded cases (i.e., 2.34 in the example), but used the p-value on the basis of the reported test statistic in other cases. We checked whether over-reported p-values were corrected upwards via procedures like Bonferroni's or Huyn-Feldt's, but did not use these corrections in analyzing p-value distributions. As some of the inconsistencies may have arisen from the use of one-sided testing, we made additional searches of the text for explicit references thereof. In one instance, an F-test result was reported explicitly as a one-sided test, but because this result was equivalent to a one-sided t-test we did not consider it erroneous (as suggested by an independent reviewer). As a final check, the three authors independently verified all 49 inconsistencies on the basis of the papers. All documented errors are available upon request.

The use of this method previously revealed quite high errors rates in the reporting of p-values in papers published in *Nature*, the *British Medical Journal* [4], and two psychiatry journals [34]. In a recent study covering a fairly representative sample of 281 psychology papers [33], roughly 50% of the papers that involved NHST were found to include at least one such reporting error. As discussed elsewhere [33], likely causes include (1) errors in the retrieval and copying of test statistics, degrees of freedom, and/or p-value (e.g., reporting the total DF instead of the error DF of an *F* test), (2) incorrect rounding of last decimal (e.g., *p* = .059 reported as *p* = .05), (3) the use of one-tailed tests without mentioning this, (4) incorrect use of tests (e.g., dividing the p value of an *F* or *χ^2^* test by two to report a one-sided *p* value, whereas the F or χ^2^ test is already a one-sided test), (5) confusion of = with<(e.g., *p* = .012 reported as *p*<.01), and (6) copy-editing errors (e.g., a failure to alter relevant numbers after the use of “copy-paste” in writing the paper). Although many inconsistencies between reported and recomputed p-values in Bakker and Wicherts' study were minor, roughly 15% of the papers contained at least one result that was presented as being statistically significant (*p*<.05), but that proved, upon recalculation, not to be significant (*p*>.05). Such serious errors in the reporting of results increase the desirability to have the data available for reanalysis.

### Ethical Considerations

This study has been approved by the Ethics Committee of the Psychology Department of the University of Amsterdam. In light of the purpose of our study, we could not ask the corresponding authors for their informed consent. The Ethics Committee exempted the use of informed consent because all corresponding authors had signed APA publication forms related to data sharing and in light of Article 8.05 of the Ethical Principles of the APA. The documented errors are based on publically available papers and so are considered archival material. The sharing or non-sharing of data is considered to be an unobtrusive observation of professional behavior of the corresponding authors that should not create distress on their behalf, provided that anonymity is assured. To protect the identity of corresponding authors, we are not allowed to make public who did or did not share data with Wicherts et al. However, this information is available upon request to allow others to verify our results through reanalysis. The problems that we highlight are general, and our results should not be used to question the academic integrity of individual researchers. The analyses we report here were all conducted independently by at least two of us on the basis of the data that all of us have in our possession.

## Results

### Responses to Data Requests

Of the 49 corresponding authors, 21 (42.9%) had shared some data with Wicherts et al. Thirteen corresponding authors (26.5%) failed to respond to the request or any of the two reminders. Three corresponding authors (6.1%) refused to share data either because the data were lost or because they lacked time to retrieve the data and write a codebook. Twelve corresponding authors (24.5%) promised to share data at a later date, but have not done so in the past six years (we did not follow up on it). These authors commonly indicated that the data were not readily available or that they first needed to write a codebook.

### Errors in the Reporting of Statistical Results

**Figure 1:**
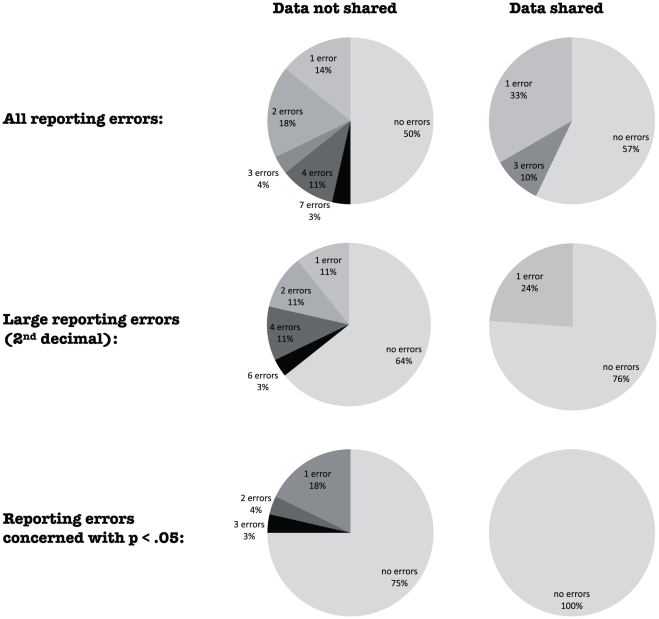
Distribution of reporting errors per paper for papers from which data were shared and from which no data were shared. Distribution of the number of errors in the reporting of p-values for 28 papers from which the data were not shared (left column) and 21 from which the data were shared (right column) for all misreporting errors (upper row), larger misreporting errors at the 2^nd^ decimal (middle row), and misreporting errors that concerned statistical significance (p<.05; bottom row).

The 49 papers contained a total of 1148 test statistics that were presented as significant at *p*<.05. Table 1 presents for each paper the number of significantly reported test results, the number of misreporting errors, and the median and average of all genuinely significant p-values (as based on the recalculated values). Forty-nine of these statistics (4.3%) were inconsistent with the reported (range of) p-values. In forty-seven of the inconsistent results (95.9%), the reported p-value (range) was smaller than the recalculated p-value. Figure 1 gives the origin of three types of reporting errors. Although 51.1% (587) of the tests statistics were from papers from which no data were shared, most incorrectly reported p-values (36 out of 49; 73.5%) originated from these papers. These errors include quite small ones (e.g., *p* = .0002 reported as *p*<.0001). Twenty-eight of the 32 p-values (87.5%) that were incorrectly reported at the level of the 2^nd^ decimal (e.g., *p* = .02 reported as *p*<.01) were from papers from which no data were shared. Negative binomial regressions (Table 2) that accounted for the number of test statistics and the average p-values in each paper (see below) showed that reluctance to share data was predictive of the prevalence of both types of reporting errors.

**Table 1:** Summary statistics for the 49 papers.

Journal	DOI	Pageno.	No. of stats.	Mean of ps	Median of ps	Reporting. errors		
						All	2nd dec.	around.05
jep∶lmc	10.1037/0278–7393.30.5.947	947–959	7	0.006636	0.00295	0	0	0
jep∶lmc	10.1037/0278–7393.30.5.969	969–987	13	0.027302	0.02936	0	0	0
jep∶lmc	10.1037/0278–7393.30.5.988	988–1001	33	0.010325	0.00482	3	0	0
jep∶lmc	10.1037/0278–7393.30.5.1002	1002–1011	25	0.004257	0.00001	1	0	0
jep∶lmc	10.1037/0278–7393.30.5.1012	1012–1025	83	0.003054	0.00000	0	0	0
jep∶lmc	10.1037/0278–7393.30.5.1026	1026–1044	30	0.007286	0.00189	0	0	0
jep∶lmc	10.1037/0278–7393.30.5.1045	1045–1064	19	0.005587	0.00073	0	0	0
jep∶lmc	10.1037/0278–7393.30.5.1065	1065–1081	22	0.001672	0.00009	3	0	0
jep∶lmc	10.1037/0278–7393.30.5.1082	1082–1092	9	0.001089	0.00010	0	0	0
jep∶lmc	10.1037/0278–7393.30.5.1093	1093–1105	21	0.011132	0.00115	1	1	0
jep∶lmc	10.1037/0278–7393.30.5.1106	1106–1118	16	0.002213	0.00001	2	2	1
jep∶lmc	10.1037/0278–7393.30.5.1119	1119–1130	10	0.007128	0.00095	0	0	0
jep∶lmc	10.1037/0278–7393.30.5.1131	1131–1142	21	0.003256	0.00098	0	0	0
jep∶lmc	10.1037/0278–7393.30.6.1147	1147–1166	8	0.008461	0.00036	0	0	0
jep∶lmc	10.1037/0278–7393.30.6.1167	1167–1175	8	0.011841	0.00231	0	0	0
jep∶lmc	10.1037/0278–7393.30.6.1176	1176–1195	32	0.005418	0.00006	1	0	0
jep∶lmc	10.1037/0278–7393.30.6.1196	1196–1210	37	0.004050	0.00000	1	1	0
jep∶lmc	10.1037/0278–7393.30.6.1211	1211–1218	11	0.019460	0.01967	0	0	0
jep∶lmc	10.1037/0278–7393.30.6.1219	1219–1234	39	0.016008	0.01084	7	6	1
jep∶lmc	10.1037/0278–7393.30.6.1235	1235–1251	23	0.004993	0.00096	1	1	0
jep∶lmc	10.1037/0278–7393.30.6.1252	1252–1270	46	0.010496	0.00058	0	0	0
jep∶lmc	10.1037/0278–7393.30.6.1271	1271–1278	20	0.002645	0.00001	1	0	0
jep∶lmc	10.1037/0278–7393.30.6.1290	1290–1301	35	0.013469	0.00475	0	0	0
jep∶lmc	10.1037/0278–7393.30.6.1302	1302–1321	30	0.013727	0.00680	0	0	0
jep∶lmc	10.1037/0278–7393.30.6.1322	1322–1337	37	0.006148	0.00094	0	0	0
jpsp	10.1037/0022–3514.87.5.557	557–572	33	0.016946	0.01104	1	1	0
jpsp	10.1037/0022–3514.87.5.573	573–585	15	0.011696	0.00597	1	0	0
jpsp	10.1037/0022–3514.87.5.586	586–598	21	0.019989	0.01519	4	4	3
jpsp	10.1037/0022–3514.87.5.599	599–614	24	0.009036	0.00263	0	0	0
jpsp^2^	10.1037/0022–3514.87.5.615	615–630	27	0.003605	0.00000	3	0	0
jpsp	10.1037/0022–3514.87.5.631	631–648	6	0.008074	0.00385	0	0	0
jpsp	10.1037/0022–3514.87.5.649	649–664	16	0.012216	0.00510	4	4	0
jpsp	10.1037/0022–3514.87.5.665	665–683	23	0.016715	0.00179	2	1	1
jpsp	10.1037/0022–3514.87.6.733	733–749	24	0.023442	0.02068	2	2	2
jpsp^1^	10.1037/0022–3514.87.6.750	750–762	5	0.000002	0.00000	0	0	0
jpsp	10.1037/0022–3514.87.6.763	763–778	29	0.007420	0.00005	1	1	0
jpsp^2^	10.1037/0022–3514.87.6.779	779–795	9	0.025925	0.03231	0	0	0
jpsp^1^	10.1037/0022–3514.87.6.796	796–816	15	0.006438	0.00072	0	0	0
jpsp	10.1037/0022–3514.87.6.817	817–831	20	0.007695	0.00011	0	0	0
jpsp	10.1037/0022–3514.87.6.832	832–844	8	0.021422	0.02079	4	4	1
jpsp	10.1037/0022–3514.87.6.845	845–859	48	0.009394	0.00380	2	0	0
jpsp	10.1037/0022–3514.87.6.860	860–875	28	0.019047	0.01104	0	0	0
jpsp	10.1037/0022–3514.87.6.876	876–893	27	0.011934	0.00598	1	1	1
jpsp^2^	10.1037/0022–3514.87.6.894	894–912	8	0.009142	0.00092	0	0	0
jpsp	10.1037/0022–3514.87.6.913	913–925	7	0.018208	0.00783	0	0	0
jpsp	10.1037/0022–3514.87.6.926	926–939	9	0.011442	0.01224	0	0	0
jpsp	10.1037/0022–3514.87.6.940	940–956	36	0.009620	0.00314	2	2	0
jpsp	10.1037/0022–3514.87.6.957	957–973	45	0.006310	0.00020	0	0	0
jpsp	10.1037/0022–3514.87.6.974	974–990	30	0.018801	0.01527	1	1	0

Note: Ethical considerations preclude the inclusion of “shared vs. non-shared” in this table, but this information is available upon request. JEP∶LMC vol. 30, JPSP vol. 87.

1 correlational design;

2 mixed correlational/experimental design, remaining papers involve experimental designs.

**Table 2:** Results of negative binomial regressions of the number of reporting errors per paper.

Predictor	Parameter (SE)	Wald χ^2^ (DF = 1)	p
All reporting errors (range: 0–7)			
(Intercept)	−2.76 (1.30)	4.53	.033
Data shared (1) or not (0)	−0.83 (0.38)	4.84	.028
Square root (Average of p-values)	4.39 (6.13)	0.51	.473
Log (No. of test statistics)	0.85 (0.41)	4.19	.041
Neg.Binomial parameter	0.83 (0.46)		
Large reporting errors at the second decimal (range: 0–6)			
(Intercept)	−4.10 (1.78)	5.30	.021
Data shared (1) or not (0)	−1.20 (0.52)	5.39	.020
Square root (Average of p-values)	17.17 (9.42)	3.32	.069
Log (No. of test statistics)	0.71 (0.45)	2.53	.112
Neg.Binomial parameter	1.41 (0.84)		

Negative binomial regressions (N = 49; with a log link) of the number of misreporting errors per paper on the log of the number of test statistics, square root of the average p-value of genuinely significant results within the papers, and whether or not the data were shared for reanalysis. Analyses were estimated in SPSS 18.0 (The Zelig package in R gave similar results) with a robust variance estimator to deal with the possibility that errors were dependent within papers. Natural log and square root transformations were used to improve predictors' normality.

We came across a total of ten cases (from seven papers) in which the recomputed p-value was above .05, whilst the result was presented as being significant (*p*<.05). None of the authors of these papers had shared data with Wicherts et al. As a negative binomial regression is infeasible with these data, we tested this relation at the level of papers (includes serious error(s) versus shared) with a 2-by-2 Fisher exact test: *p* = .015 (2-tailed). So the reluctance to share data was particularly evident when the reporting errors concern statistical significance. This corroborates an earlier finding that it took authors considerably longer to respond to queries for data when the inconsistency in their reported results had a bearing on the significance of their results [33].

### Strength of Evidence (against the Null Hypothesis)

**Figure 2:**
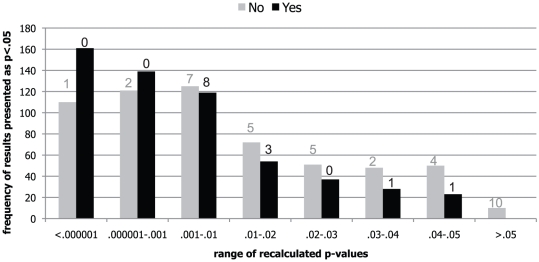
Distribution of p-values reported as being significant in papers from which data were shared or not. Distribution of p-values reported as being significant (at p<.05) in 21 papers from which data were shared (N = 561; in black) and in 28 papers from which data were not shared (N = 587; in grey), showing that p-values often lie closer to the typical boundary of significance when data are not shared for reanalysis. Frequencies of reporting errors (as given above the bars) reflect higher error prevalence in papers from which no data were shared.

P-values from NHST are traditionally interpreted as the strength of the evidence against the null hypothesis of no effect [31]. From the distribution of significant p-values across the two groups of papers in Figure 2, it is clear that higher p-values, like those in the interval between .03 and .05 (which have a low chance of occurring regardless of actual effect sizes [35]), were indeed more common in papers from which no data were shared (16.7%) than in the other papers (9.1%). The individual statistical results are statistically dependent within papers in rather intractable ways, and so we computed the mean of p-values of all genuinely significant results within each paper. This variable was indeed significantly higher in the 28 papers from which the data were not shared (M = .0124, SD = .0074, Median = .0114 vs. M = .0079, SD = .0046, Median = .0073, Cohen's d = .72): Wilcoxon's *W* = 413, *Z* = 2.26, *p* = .024 (2-tailed). The use of the median of p-values per paper provided similar results (*Z* = 2.14, *p* = .032).

We also conducted a bootstrap analysis to test this difference between shared and non-shared papers on the basis of individual p-values as clustered in the papers. In this analysis, we determined on the basis of 100,000 replications the null distribution of Wilcoxon's W test for the 1138 statistically dependent p-values that were smaller than .05. To this end, we randomly assigned each paper (and hence all p-values in it) to either the shared or non-shared category (on the basis of the base rate of p = 21/49), and repeated this process 100,000 times to get an empirical null distribution for W on the basis of our data. The W statistic computed on the basis of actual difference between shared and non-shared gave a p-value of .0298 (2-tailed) in this bootstrapped null distribution. Hence, the analyses of individual p-values corroborated that p-values were significantly higher in papers from which data were not shared.

## Discussion

In this sample of psychology papers, the authors' reluctance to share data was associated with more errors in reporting of statistical results and with relatively weaker evidence (against the null hypothesis). The documented errors are arguably the tip of the iceberg of potential errors and biases in statistical analyses and the reporting of statistical results. It is rather disconcerting that roughly 50% of published papers in psychology contain reporting errors [33] and that the unwillingness to share data was most pronounced when the errors concerned statistical significance. Although our results are consistent with the notion that the reluctance to share data is generated by the author's fear that reanalysis will expose errors and lead to opposing views on the results, our results are correlational in nature and so they are open to alternative interpretations. Although the two groups of papers are similar in terms of research fields and designs, it is possible that they differ in other regards. Notably, statistically rigorous researchers may archive their data better and may be more attentive towards statistical power than less statistically rigorous researchers. If so, more statistically rigorous researchers will more promptly share their data, conduct more powerful tests, and so report lower p-values. However, a check of the cell sizes in both categories of papers (see Text S2) did not suggest that statistical power was systematically higher in studies from which data were shared.

The association between reporting errors and sharing of data after results are published may also reflect differences in the rigor with which researchers manage their data. Rigorously working researchers may simply commit fewer reporting errors *because* they manage and archive their data more diligently. A recent survey among 192 Dutch psychological researchers highlighted a rather poor practice of data archiving in psychology [36]. When asked whether they archived their research data, only a third of the psychologists responded positively. This is remarkable in light of guidelines of the APA [11] that stipulate that data should be retained a minimum of five years after publication of the study. Even among those psychologists who indicated that they “archive” their data, most did not follow proper archiving standards (e.g., by keeping code books and writing meta-data [37]), but simply stored data on their own (current) computer (32%), on CDs/DVDs (18%), or on the shelf (20%). Haphazard data management is documented in a number of scientific fields [37], [38], [39], may result in errors in analyzing and reporting of results, and obviously impedes the sharing of data after results are published.

Regardless of the underlying processes, the results on the basis of the current papers imply that it is most difficult to verify published statistical results when these are contentious. We focused here on NHST within two psychology journals and so it is desirable to replicate our results in other fields and in the context of alternative statistical approaches. However, it is likely that similar problems play a role in the widespread reluctance to share data in other scientific fields [13], [14], [15], [16], [17], [18], [19], [20]. Because existing guidelines on data sharing offer little promise for improvement [40], progress in psychological science and related fields would benefit from having research data itself be part of the process of replication [15], [16], notably by the establishment by journals, professional organizations, and granting bodies of mandatory data archiving policies.

More stringent policies concerning data archiving will not only facilitate verification of analyses and corrections of the scientific record, but also improve the quality of reporting of statistical results. Changing policies require better educational training in data management and data archiving, which is currently suboptimal in many fields [36], [37], [38], [39]. On the other hand, technical capabilities for storage are already available. For instance, several trial registers in the medical sciences (like clinicaltrials.gov) enable storage of research data. Rigorous archiving of data involves documentation of variables, meta-data, saving data files in formats that are robust (e.g., ASCII files), and submitting files to repositories that already require these standards. Best practices in conducting analyses and reporting statistical results involve, for instance, that all co-authors hold copies of the data, and that at least two of the authors independently run all the analyses (as we did in this study). Such double-checks and the possibility for others to independently verify results later should go a long way in dealing with *human factors* in the conduct of statistical analyses and the reporting of results.

## Supporting Information

Text S1 **E-mail sent in 2005 to request data from published papers.**
(DOC)

Text S2 **Consideration of potential differences in statistical power between the two types of papers.**
(DOC)
