# Supplementary material for: Willingness to Share Research Data Is Related to the Strength of the Evidence and the Quality of Reporting of Statistical Results
Source: PLoS One. 2011 Nov 2;6(11):e26828. doi: 10.1371/journal.pone.0026828 (PMC3206853; doi:10.1371/journal.pone.0026828)
Supplement: Text S2 — Consideration of potential differences in statistical power between the two types of papers. (DOC) [file pone.0026828.s002.doc]

**Consideration of potential differences in statistical power between the two types of papers.**

It is possible that the studies from which data were shared have larger sample sizes than the studies from which no data were shared. This may have led to difference in statistical power and hence the difference in p-values. This possibility can be studied by comparing the papers on the basis of the typical cell size as based on the degrees of freedom from t tests and particular F tests. So we considered the denominator DFs from F tests that had DF = 1 in the numerator and the DFs of all the t tests in the papers. We computed the mean of the error DF as an indicator of typical cell size in the analyses for each of the 48 papers that reported such statistics. The mean of this variable in 27 papers that were not shared (M = 199, SD = 657, Md = 57) was somewhat higher than the mean in the 21 papers from which data were shared (M = 77, SD = 107, Md = 45), albeit not significantly so: Wilcoxon’s W = 441, *Z* = 1.53, *p* = .127 (2-tailed). Hence, the cell sizes in these analyses did not suggest that statistical power was systematically higher in studies from which data were shared.
