# Supplementary material for: Willingness to Share Research Data Is Related to the Strength of the Evidence and the Quality of Reporting of Statistical Results
Source: PLoS One. 2011 Nov 2;6(11):e26828. doi: 10.1371/journal.pone.0026828 (PMC3206853; doi:10.1371/journal.pone.0026828)
Supplement: Text S1 — E-mail sent in 2005 to request data from published papers. (DOC) [file pone.0026828.s001.doc]

**E-mail sent in 2005 to request data**

The request email was based specifically on Standard 8.14 of APA’s Ethical Principles of Psychologists and Code of Conduct (http://www.apa.org/ethics/code/index.aspx) and ran as follows:

Dear Dr. [Author’s name],

As a student at the Psychology Department of the University of Amsterdam I am currently conducting a study supervised by Drs. Wicherts and Borsboom. For this research I am gathering data from studies, which are published in several APA-journals in 2004. The aim of our study is to verify the substantive claims made in published research through reanalysis of data. Our specific aim is to assess the general impact of outliers on effect sizes and correlations reported in psychological research. Therefore I would like to ask you if you would be so kind as to send me the raw data of the [study/studies] reported in your paper ‘[paper title]’, published in [journal name]. Of course I realize that it will take some time and effort to fulfill this request. Please note, however, that it is not necessary to completely rewrite the databook. You can just send me the file with the essential data and I will contact you later if something appears to be unclear. I would greatly appreciate it if you would help me by letting me use your data. Of course, the confidentiality of the participants in your study is guaranteed. Thank you for considering my request and I am looking forward to your answer.

Yours sincerely,

Mrs. J. Kats – Psychological Methods, Department of Psychology, University of Amsterdam
